# The Biomechanics and Optimization of the Needle-Syringe System for Injecting Triamcinolone Acetonide into Keloids

**DOI:** 10.1155/2016/5162394

**Published:** 2016-10-24

**Authors:** Anthony Vo, Marc Doumit, Gloria Rockwell

**Affiliations:** ^1^Department of Diagnostic Imaging, University of Alberta, Edmonton, AB, Canada; ^2^Faculty of Mechanical Engineering, University of Ottawa, Ottawa, ON, Canada; ^3^Division of Plastic Surgery, Department of Surgery, University of Ottawa, Ottawa, ON, Canada

## Abstract

*Purpose*. Injecting triamcinolone acetonide (TA) into a keloid is physically challenging due to the density of keloids. The purpose was to investigate the effects of various syringe and needle combinations on the injection force to determine the most ergonomic combination.* Materials and Methods*. A load cell was used to generate and measure the injection force.* Phase 1: *the injection force of 5 common syringes was measured by injecting water into air. The syringe that required the lowest injection force was evaluated with various needle gauges (25, 27, and 30 G) and lengths (16, 25, and 38 mm) by injecting TA (40 mg/mL) into air. The needle-syringe combination with the lowest injection force (CLIF) was deemed the most ergonomic combination.* Phase 2:* comparisons between the CLIF and a standard combination (SC) were performed by injecting TA into air and tap water into a keloid specimen. Intraclass Correlation Coefficient (ICC) and independent *t*-test were used. * Results*. Increasing the syringe caliber, injection speed, and needle gauge and length significantly increased the injection force (*p* value < 0.001). The SC required a maximum force of 40.0 N to inject water into keloid, compared to 25.0 N for the CLIF. Injecting TA into keloid using the SC would require an injection force that was 103.5% of the maximum force female thumbs could exert compared to 64.8% for the CLIF. ICC values were greater than 0.4.* Conclusions*. The 1 mL polycarbonate syringe with a 25 G, 16 mm needle (CLIF) was the most ergonomic combination. The SC required a substantial injection force, which may represent a physical challenge for female thumbs.

## 1. Introduction 

A keloid scar represents an overly aggressive response to wound healing. Histologically, it is characterized by the presence of large, dense, and broad collagen fibers arranged in nodular formations [1]. On examination, it is seen as an elevated fibrous scar that extends beyond the original injury site, which does not regress with time. It can be accompanied by itchiness, pain, restricted mobility, and disfiguring dermatoses. Therefore, patients can have severe psychological and physical impairments [2].

The current standard for managing a keloid is a series of synthetic corticosteroid injections intralesionally, often triamcinolone acetonide (TA). Injecting into the lesion is often difficult because a substantial amount of injection pressure is required to deliver the medication into the dense keloid. Compared to normal unscarred skin, keloids have collagen synthesis and breakdown that are 20 times and 14 times greater, respectively [1]. Consequently, an important factor for the success of an injection is the ability of the physician to comfortably generate an injection pressure sufficient to overcome this resistance [3].

The pressure that can be generated for any particular syringe at a predetermined speed depends on the force exerted by the physician divided by the surface area of the syringe plunger (pressure_generated_ = force_thumb_/area_syringe  plunger_). An injection is conventionally performed with the thumb pushing on the plunger while the ipsilateral index and middle fingers are used to stabilize the syringe flanks. In this particular position, the average maximum force that can be generated is 79.8 N (males: 95.4 N, females: 64.1 N) [4]. This force is dependent on the operator's upper limb musculature and will vary among physicians. Although this cannot be controlled, several other factors can be.

By choosing a smaller syringe caliber, which consists of a smaller surface area of its syringe plunger, the same injection force can generate a higher pressure. Or, more significantly, a lower injection force can generate the same pressure with a smaller syringe caliber. In practice, this can be achieved by choosing a smaller syringe volume, which is acceptable considering 0.1–0.5 mL is often injected per square centimeter of the keloid [5]. A smaller syringe volume will also provide enhanced needle control over larger syringes when performing procedures [6]. Therefore, it is important to determine an ergonomically feasible syringe volume that will allow a physician to use less force to inject into keloids.

Another factor that needs to be considered is the needle. High needle gauges are recommended for managing keloids because they inflict less pain [3, 7]. However, they are more likely to become occluded when a dense injectant such as TA is used. Moreover, the smaller needle orifice will yield a higher flow resistance and consequently a larger injection force. The length of the needle must also be considered because it contributes to the flow resistance. Therefore, the bigger the gauge and the longer the length of the needle, the higher the injection force required by the physician.

Thus, the combination of syringe and needle can be optimized to reduce the injection force required to inject TA into a keloid. It is therefore important to determine the most ergonomic combination of syringe and needle. From an author's experience (GR), a 3 mL polypropylene syringe with a 25 G, 16 mm needle is the standard combination (SC) used at her institution to deliver TA into keloids.

A review of the literature revealed that there is no scholarly work studying the effects of various syringe and needle parameters within the context of keloid management (speed of injection, needle length and gauge, etc.). Furthermore, there is currently no consensus as to which needle-syringe combination is the most ergonomic for injecting TA into keloids. The purpose of this study was to investigate the effects of various syringe and needle combinations on the force of injection of TA, in order to determine the most ergonomic combination for injecting TA into keloids.

## 2. Materials and Methods

### 2.1. Materials

**Figure 1:**
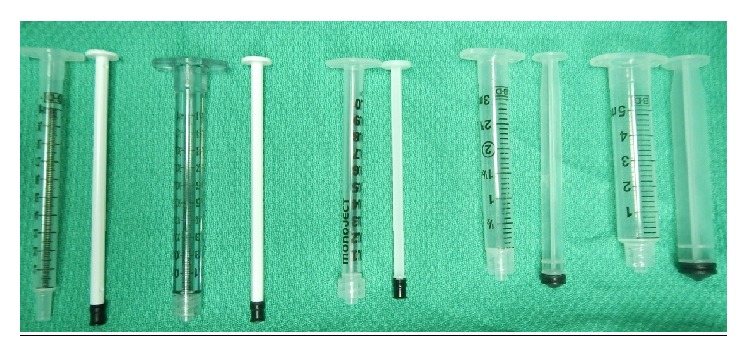
Five syringes identified for the study. From left to right: 1 mL Tuberculin polypropylene, 1 mL polycarbonate, 1 mL Monojet polypropylene, 3 mL polypropylene, and 5 mL polypropylene.

Five syringes were identified for this study (see Table 1 and Figure 1). Insulin syringes (BD, New Jersey, USA) were excluded because they were manufactured with stock needles that could not be altered. Three common needle gauges were studied. The length of the 27 G and 30 G needles was 13 mm. The lengths of the 25 G needles were 16, 25, and 38 mm; 13 mm was not manufactured (see Table 1). The injectant used included room air, tap water, and 40 mg/mL TA suspension (Cytex Pharmaceuticals Inc., Halifax, Canada).

### 2.2. Methods

**Figure 2:**
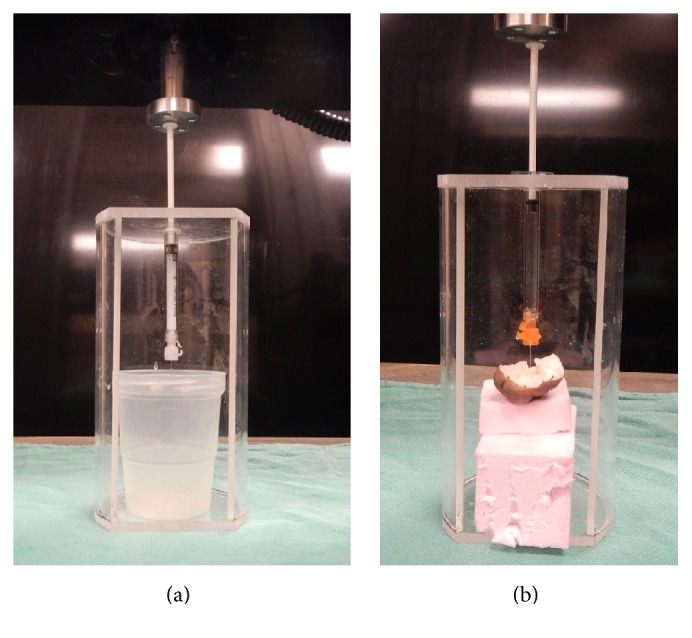
Experimental setup. 1 mL polycarbonate syringe is securely mounted with a 100 N load cell engaging the syringe plunger at 1 mm/sec. Injecting triamcinolone acetonide without backpressure (a) and injecting tap water into a keloid sample at a depth of 7 mm (b).

The injection force for various syringe and needle combinations was carried out by the experimental setup shown in Figures 2(a) and 2(b). An apparatus was created to restrain the syringe vertically under an Instron Bluehill 4482 tensile testing machine (Instron, MA, USA). The machine was programed to push and displace the syringe plunger at a constant speed. The Instron machine-loading cell measured the injection force subjected to the plunger with a maximum capacity of 100 N. The plunger displacement and injection force measurements were recorded every 0.1 second using a data acquisition system.

#### 2.2.1. Phase 1

The injection force of the five syringes was determined at three predetermined injection speeds (1, 3, and 5 mm/sec) by injecting tap water into air. 25 G, 16 mm needles were attached to the syringes to create a constant and reproducible flow resistance in order to accentuate the differences between the syringes.

The syringe with the lowest injection force was determined by analyzing the results at 1 mm/sec, the speed at which a medication is manually delivered into a patient with a syringe [8]. To determine the best needle for this syringe, the needle gauge and length were evaluated. Various needle gauges (25, 27, and 30 G) were attached to the syringe and the injection force was determined by injecting TA into air at 1 mm/sec. The optimal needle gauge was defined as the gauge that was occluded the least by TA and required the lowest injection force. Once the optimal needle gauge was determined, the length of the needle was varied and the injection force required to inject TA at 1 mm/sec into air was determined for each needle length. The combination of syringe and needle gauge and length requiring the lowest injection force (CLIF) was deemed the most ergonomic combination. Using this combination, the injection force of TA at the three injection speeds was determined.

#### 2.2.2. Phase 2

The CLIF was then compared to the SC. The injection force required to inject TA into air at 1 mm/sec was measured using the SC. In order to compare their injection force within a clinical context, a keloid sample was used. The sample was obtained from a male donor who underwent an excision of an ear keloid, followed by radiation therapy. Half of the sample was sent for standard pathology and the remaining half was stored in standard formaldehyde solution for the study. Each combination infiltrated the keloid sample at a virgin site and at a depth of 7 mm, the maximum depth often used for intralesional keloid injections [3]. The force required to inject tap water into the keloid at 1 mm/sec was measured. Results were discarded if the experiment did not run as described (e.g., not at the correct depth, injectant leaking out of the keloid).

### 2.3. Statistical Analyses

Analyses were performed using SPSS software package (version 20; SPSS, Chicago, Illinois). Descriptive statistics included the average injection force, standard deviation, and maximum injection force. Statistical significance was determined by independent *t*-test, with statistical significance defined as a *p* value less than 0.05. Intraclass Correlation Coefficient (ICC) was used to determine experimental reliability.

## 3. Results

### 3.1. Phase 1

**Figure 3:**
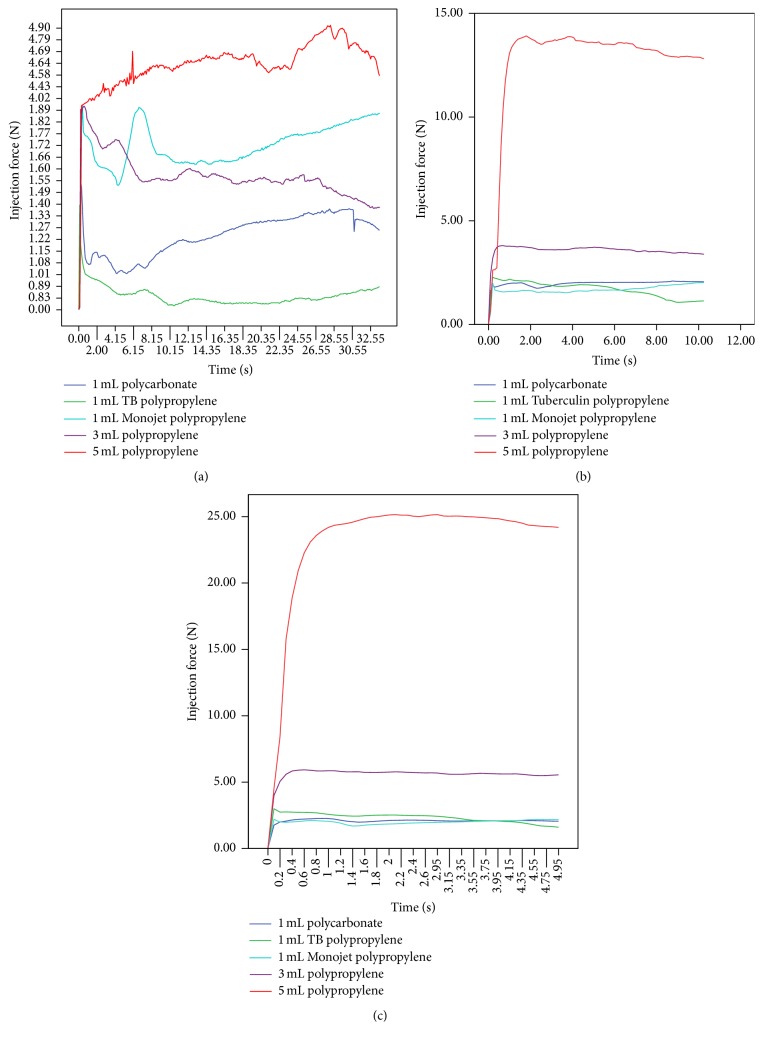
Injection force required for tap water in 5 different syringes with 25 G, 16 mm needles at 1 mm/sec (a), 3 mm/sec (b), and 5 mm/sec (c).

The results for the 5 syringes at the three predetermined injection speeds are presented in Table 2 and Figure 3. The 1 mL Tuberculin polypropylene syringe was not chosen as the syringe with the lowest injection force because it lacked a Luer-Lok tip to prevent separation of the needle from the syringe, which occurred when an average injection force greater than 15 N was applied. Instead, the 1 mL polycarbonate syringe was chosen, which required an average injection force of 1.2 N.

When the results of the 1, 3, and 5 mL polypropylene syringes with 25 G, 16 mm needles were compared, the average injection forces of the 3 and 5 mL syringes were significantly greater than the 1 mL at all three injection speeds. The 5 mL syringe required 5.3, 7.7, and 10.2 times more force for injection at 1, 3, and 5 mm/sec, respectively (see Table 3).

**Figure 4:**
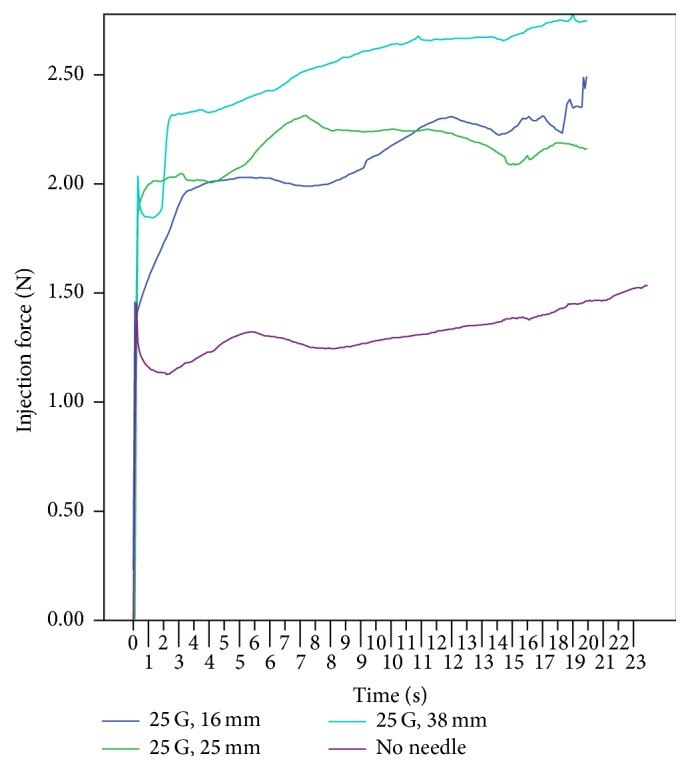
Relationship between injection force of triamcinolone acetonide and length of the 25 G needle using a 1 mL polycarbonate syringe at injection speed of 1 mm/sec.

Increasing the needle gauge significantly increased the probability of TA occluding the needle and the average injection force required. The probability of successfully injecting a full syringe volume of TA into air without occlusion was 66.7%, 15.4%, and 0% for 25, 27, and 30 G needles, respectively. The force to inject a 30 G needle was 2.9 times higher than the 25 G needle. When the length of the needle increased from 16 to 25 mm, the average injection force increased by 0.1 N. From 16 to 38 mm, the average force increased by 1.2 N (see Figure 4). Therefore the 1 mL polycarbonate syringe with a 25 G, 16 mm needle was considered the CLIF and was deemed the most ergonomic combination.

**Figure 5:**
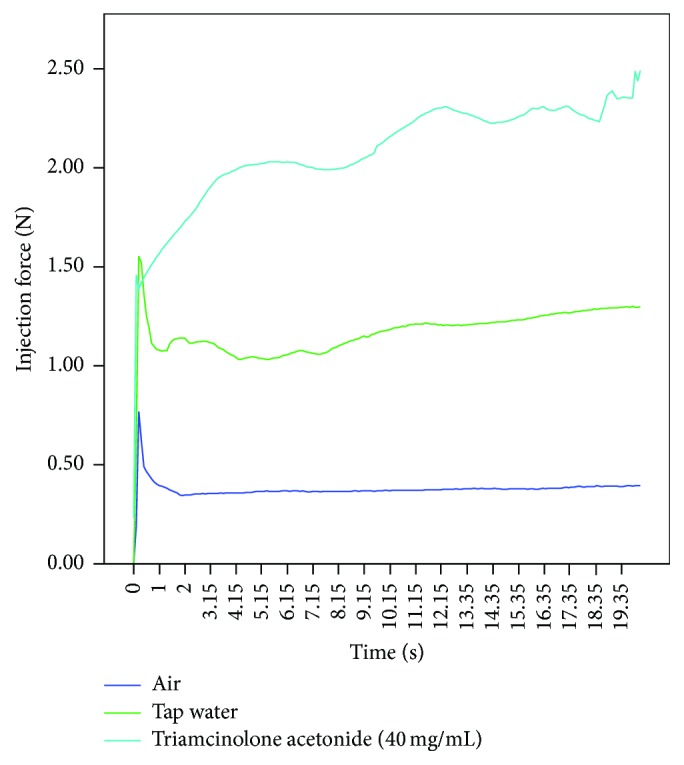
Injection force required for the different injectants using a 1 mL polycarbonate syringe with a 25 G, 16 mm needle at injection speed of 1 mm/sec.

Using the CLIF, the average force required to inject TA into air at 1 mm/sec was 2.0 N and 3.6 N at 5 mm/sec (see Table 4). Compared to tap water, injecting TA into air required 1.7 times more force (see Figure 5).

### 3.2. Phase 2

When water was injected into the keloid sample at 1 mm/sec, the CLIF required an average injection force of 17.0 N ± 6.3. The SC required an average injection force of 33.5 N ± 8.7. The maximum injection force for the CLIF was 25.0 N, compared to 40.0 N for the SC. With TA, the injection force would require 41.5 N and 66.2 N for the CLIF and the SC, respectively.

All experiments had an ICC value greater than or equal to 0.4, indicating being moderately reliable or better. All experimental differences compared were statistically significant (*p* < 0.001).

## 4. Discussion

### 4.1. Effects of Syringe Caliber and Injection Speed on the Injection Force

Comparing the three syringe volumes (1, 3, and 5 mL) made with the same material (polypropylene) and manufacturer, the syringe caliber significantly affected the injection force required. At the highest injection speed (5 mm/sec), the 5 mL syringe required an average of 23.3 N, which was 10.2 times higher than the 1 mL syringe (2.1 N). These findings were expected because the injection force is a function of several factors, namely, (1) overcoming the resistance of the plunger, (2) imparting kinetic energy to the injectant, (3) forcing the injectant through the needle, and (4) exceeding the backpressure of the injection site [9]. In this particular experiment, factors 1 and 4 were negligible. Increasing the syringe caliber by choosing a bigger syringe volume increases the surface area of the syringe plunger, resulting in a greater surface contact with tap water. In order to displace the greater volume of tap water at the predetermined injection speed through a constant needle orifice, a greater force is needed. Similarly, increasing the kinetic energy of the injectant will require a greater injection force. These findings are consistent with results from a previous study [10]. Therefore, it is important to select a small syringe caliber and to inject at a low speed.

### 4.2. Effects of Needle Gauge and Length on the Injection Force

Increasing the needle gauge significantly increased the injection force and the probability of the needle being occluded by the injectant. A 27 and 30 G needle had significantly higher chance of being occluded by TA suspension (40 mg/mL) than a 25 G needle. In a study performed by Cilurzo et al., they concluded that the only significant parameter influencing the extrusion of the injectant through a given needle-syringe system was the gauge of the needle [8]. The authors of this paper recommend 25 G needles.

Adjusting the length of a 25 G needle from 16 to 25 and 38 mm increased the injection force of TA. In a similar study, the force required to sustain the movement of the plunger proportionally increased with respect to the needle length [8]. By increasing the length of the needle, the flow resistance increases, requiring a higher injection force. Therefore, a 16 mm needle length is recommended. This needle length is sufficient since most intralesional injections are done at a depth of 3 to 7 mm [3].

### 4.3. Effects of Injectants on the Injection Force

Increasing the density of the injectant significantly increased the force of injection as expected. At 1 mm/sec, the injection force progressively increased from 0.5 N to 1.2 N and to 2.2 N when room air, tap water, and TA were injected into air, respectively. TA required 1.7 times more force than tap water to inject. By reducing the concentration of TA (e.g., diluting), a lower injection force may be achieved.

### 4.4. Effects of the Keloid Specimen on the Injection Force

TA was originally used for injection into the keloid, but it was stopped. It consistently occluded the needle when each experiment was being prepared, which required the needle to be exchanged and reinfiltrated into the keloid, risking the integrity of the one keloid sample available. Therefore, tap water was used as the injectant. With tap water, only a limited number of experiments could be performed before the keloid was saturated, hydrodissected, and severely infiltrated. Infiltration was done at a depth of 7 mm to simulate a clinical encounter.

The SC required an average injection force that was 1.6 times higher than the CLIF, when tap water was injected into the keloid at 1 mm/sec. For males, the CLIF required 26.3% of the maximum force male thumbs could exert compared to 41.9% for the SC. For females, the CLIF required 39.0% compared to 62.3% for the SC.

Based on the experimental results from injecting into air, a TA injection into a keloid would require an injection force that is 1.7 times more than tap water. For females, the SC requires a maximum injection force that is 103.5% of the maximum force female thumbs can exert, compared to 64.8% for the CLIF. For males, the SC requires 69.2% compared to 43.6% for the CLIF [4]. Therefore a physician, especially a female, is more likely to generate a sufficient injection force with the CLIF than the SC. With repeated injections, the SC may more likely cause thumb discomfort and fatigue, regardless of gender.

The caliber of the syringe can largely explain the difference in the injection force between the two combinations. The CLIF's 1 mL syringe has a caliber that is 3.3 mm and a surface area of 81.5 mm^2^ compared to a caliber of 59.6 mm and a surface area of 27513.0 mm^2^ from SC's 3 mL syringe (see Table 5). Therefore, to generate a sufficient pressure that overcomes the resistance of a keloid, the CLIF syringe would require a lower injection force. However, due to the complex shape of the syringe and needle, it is difficult to precisely quantify the effects of syringe caliber and surface area without deviations and therefore only qualitative comparisons can be made.

### 4.5. Limitations and Future Directions

The manufacturer of the needles did not produce 25 G, 13 mm needles to allow accurate comparisons between the gauges. However, the authors feel that this did not affect the results and interpretations made. Furthermore, the heterogeneity of the biologic keloid sample requires a large sample size to increase reliability. Additional keloid samples would strengthen this study. Lastly, the findings of this study could be extrapolated to other clinical scenarios requiring needle injections. For example, in situations where a high volume of injectant is required, an ergonomic needle-syringe combination would reduce the injection force required by a physician to achieve the same injection pressure. This would reduce mechanical stress and fatigue on the physician.

## 5. Conclusion

A particular needle-syringe combination can significantly affect the injection force a physician has to generate to perform an injection. Increasing the needle gauge and syringe caliber significantly affected the success of an injection. The success rates of injecting TA using 27 and 30 G needles were poor due to occlusion of the needle. Extrapolations may be made to other types of injections (e.g., local anesthesia). In this study, the 1 mL polycarbonate syringe with a 25 G, 16 mm needle (CLIF) was the most ergonomic combination for injecting into keloids. The standard combination (SC) required a substantial injection force, which may represent a physical challenge for those with lesser thumb strength, especially females.

## Figures and Tables

**Table 1:** Syringe and needle materials investigated.

Syringes (manufacturer)	1 mL polycarbonate (Becton, Dickinson and Company, New Jersey, USA)	1 mL Monojet polypropylene (Tyco Healthcare Group, Massachusetts, USA)	1 mL Tuberculin polypropylene (Becton, Dickinson and Company, New Jersey, USA)	3 mL polypropylene (Becton, Dickinson and Company, New Jersey, USA)	5 mL polypropylene (Becton, Dickinson and Company, New Jersey, USA)

Needle lengths and gauges (manufacturer)	16 mm, 25 G BD Eclipse needles (Becton, Dickinson and Company, New Jersey, USA)	25 mm, 25 G BD Eclipse needles (Becton, Dickinson and Company, New Jersey, USA)	38 mm, 25 G BD Eclipse needles (Becton, Dickinson and Company, New Jersey, USA)	13 mm, 27 G BD Eclipse needles (Becton, Dickinson and Company, New Jersey, USA)	13 mm, 30 G BD Eclipse needles (Becton, Dickinson and Company, New Jersey, USA)

**Table 2:** The average injection force, standard deviation, maximum injection force, Intraclass Correlation Coefficient (ICC), *p* value, and *p* value comments for the 5 syringes with 25 G, 16 mm needles injecting tap water at 3 different injection speeds into air.

	Average force (N) ± standard deviation	Maximum force (N)	ICC	*p* value	Comparison for *p* value
*25 G, 16 mm needle with water at 1 mm/sec*					
1 mL polycarbonate (A)	1.6	0.6	0.6		
1 mL Monojet polypropylene	2.2	0.5	0.5	*p* < 0.001	Compared to A
1 mL Tuberculin polypropylene	1.4	0.4	0.4	*p* < 0.001	
3 mL polypropylene	1.9	0.9	0.9	*p* < 0.001	
5 mL polypropylene	4.9	0.6	0.6	*p* < 0.001	
*25 G, 16 mm needle with water at 3 mm/sec*					
1 mL polycarbonate (B)	1.9 ± 0.3	2.1	0.8	*p* < 0.001	Compared to A
1 mL Monojet polypropylene	1.7 ± 0.3	2.2	0.8	*p* < 0.001	Compared to B
1 mL Tuberculin polypropylene	1.6 ± 0.5	2.3	0.9	*p* < 0.001	
3 mL polypropylene	3.5 ± 0.6	3.8	1	*p* < 0.001	
5 mL polypropylene	12.4 ± 3.2	13.9	0.8	*p* < 0.001	
*25 G, 16 mm needle with water at 5 mm/sec*					
1 mL polycarbonate (C)	2.1 ± 0.3	2.3	1	*p* < 0.001	Compared to A
1 mL Monojet polypropylene	2.0 ± 0.2	2.7	0.9	*p* < 0.001	Compared to C
1 mL Tuberculin polypropylene	2.3 ± 0.4	3	1	*p* < 0.001	
3 mL polypropylene	5.5 ± 0.7	6	0.8	*p* < 0.001	
5 mL polypropylene	23.3 ± 4.4	25.2	1	*p* < 0.001	

**Table 3:** Ratios of injection force required for 3 and 5 mL polypropylene syringes relative to 1 mL Tuberculin polypropylene syringe at 1, 3, and 5 mm/sec, using 25 G, 16 mm needles.

	1 mm/sec	3 mm/sec	5 mm/sec
3 mL polypropylene	1.8x	2.2x	2.4x
5 mL polypropylene	5.3x	7.7x	10.2x

**Table 4:** The average injection force, standard deviation, maximum injection force, Intraclass Correlation Coefficient (ICC), *p* value, and *p* value comments for 1 mL polycarbonate syringes with 25 G, 16 mm needles injecting triamcinolone acetone (40 mg/mL) at 3 injection speeds into air.

	Average force (N) ± standard deviation	Maximum force (N)	ICC	*p* value	Comparison for *p* value
1 mm/sec (A)	2.0 ± 0.3	2.6	0.5		
3 mm/sec	2.9 ± 0.4	3.6	0.4	*p* < 0.001	Compared to A
5 mm/sec	3.6 ± 0.7	4.1	0.8	*p* < 0.001	

**Table 5:** Measured syringe caliber and surface area of syringe plungers.

	Syringe caliber (mm)	Measured surface area (mm^2^)
1 mL polycarbonate	3.3	81.5
1 mL Monojet polypropylene	3.3	81.5
1 mL Tuberculin polypropylene	3.3	81.5
3 mL polypropylene	59.6	27513
5 mL polypropylene	77	45964
